# Eco-Friendly Fluorine Functionalized Superhydrophobic/Superoleophilic Zeolitic Imidazolate Frameworks–Based Composite for Continuous Oil–Water Separation

**DOI:** 10.3390/molecules28062843

**Published:** 2023-03-21

**Authors:** Wenlong Xiang, Siyu Gong, Jiabin Zhu

**Affiliations:** 1 College of Chemistry, Chemical Engineering and Environment, Minnan Normal University, Zhangzhou 363000, China; 2 Fujian Province University Key Laboratory of Pollution Monitoring and Control, Minnan Normal University, Zhangzhou 363000, China

**Keywords:** oil/water separation, oil collection, metal-organic frameworks, composite sponge, superhydrophobic, green modification

## Abstract

Superhydrophobic metal−organic framework (MOF)-based sponges have received increasing attention in terms of treating oil−water mixtures. However, highly fluorinated substances, commonly used as modifiers to improve the hydrophobicity of MOFs, have aroused much environmental concern. Developing a green hydrophobic modification is crucial in order to prepare superhydrophobic MOF-sponge composites. Herein, we report the preparation of a porous composite sponge via a polydopamine (PDA)-assisted growth of zeolitic imidazolate frameworks (ZIF-90) and eco-friendly hydrophobic short-chain fluorinated substances (trifluoroethylamine) on a melamine formaldehyde (MF) sponge. The composite sponge (F-ZIF-90@PDA-MF) exhibited superhydrophobicity (water contact angle, 153°) and superoleophilicity (oil contact angle, 0°), which is likely due to the combination of the low surface energy brought on by the grafted CF_3_ groups, as well as the rough surface structures that were derived from the in situ growth of ZIF-90 nanoparticles. F-ZIF-90@PDA-MF showed an excellent adsorption capacity of 39.4–130.4 g g^−1^ for the different organic compounds. The adsorbed organic compounds were easily recovered by physical squeezing. Continuous and selective separation for the different oil−water mixtures was realized by employing the composite sponge as an absorbent or a filter. The separation efficiency and flux reached above 99.5% and went up to 7.1 ×10^5^ L m^−2^ h^−1^, respectively. The results illustrate that the superhydrophobic and superoleophilic F-ZIF-90@PDA-MF sponge has potential in the field of water−oil separation, especially for the purposes of large-scale oil recovery in a water environment.

## 1. Introduction

The rapid increase in organic compound pollution produced by increased oil leakage and the petrochemical industry has become one of the top environmental concerns worldwide [1,2]. Adsorption technology, as a versatile type of technology, has been widely applied to efficiently separate oil from water and for the purpose of organic compound recovery [3]. Improving the selective absorption and absorption capacities of adsorbents is essential. Various oil absorption materials have been widely developed, including inorganic mineral clays and fibers, as well as aerogel and commercial sponge-based materials [4,5,6,7,8]. Among them, commercial sponge-based materials have gained considerable interest because of their outstanding advantages, including high adsorption capacity, excellent mechanical properties, and low cost. Recently, superhydrophobic/superoleophilic materials have been considered suitable for selective oil−water separation [9,10]. Previous studies have demonstrated that superhydrophobic oil−water separation materials can be fabricated from commercial sponges as ideal substrates [7,11,12]. Moreover, the easy functionalization of commercial sponges can significantly improve their hydrophobicity [10,13,14]. There are two acknowledged determining factors in achieving a superhydrophobic surface, which are found in a chemical composition with low surface energy and a rough structure [12]. As porous crystalline materials, metal−organic frameworks (MOFs) have received growing attention worldwide due to their distinctive properties, such as their rich functionalities and high internal surface areas [15,16]. MOFs and their derivative materials with unique hydrophobic properties provide new ideas in different application fields [17,18,19,20,21], including in oil−water separation. Many studies confirm that hydrophobicity is a crucial factor in the applications of MOFs [22,23]. Highly hydrophobic MOF-based sponge materials have recently been developed to efficiently separate oil−water mixtures [24,25,26]. A common and effective preparation strategy involves the collaborative design of high roughness and low surface energy [27]. However, a handful of MOFs has been described as hydrophobic among the over 20 000 MOFs reported [19]. To improve the hydrophobicity of MOFs, the grafting of hydrophobic molecular modifiers on their frameworks is an effective approach. Highly fluorinated substances are one of the more common hydrophobic modifiers that are used to reduce surface energy [28,29,30,31,32]. However, the use of long-chain perfluorinated chemicals has aroused serious environmental concerns regarding the potential adverse health effects [33,34,35]. Currently, fluorine-carbon molecules with shorter chains are the most developed substituents [36,37].

Zeolitic imidazolate framework-90 (ZIF-90), one of the most typical MOFs, contains free aldehyde groups in its frameworks and thus provides a platform for post-synthetic modifications via the reaction between aldehyde groups and amine groups. It has been demonstrated that this strategy effectively enhances its physical and chemical properties [38,39]. Incorporation of short fluorine-carbon chain substances that contain amine groups in ZIF-90 via a Schiff base condensation reaction is likely a promising method to improve its hydrophobicity. The trifluoromethyl (CF_3_) group has been widely used in previous studies to increase the hydrophobicity of materials due to its extremely low surface energy [40,41]. Thus, eco-friendly short-chain fluorinated molecules containing NH_2_ groups such as 2,2,2-trifluoroethylamine should be seriously considered to improve the hydrophobicity of ZIF-90. Until now, few reports have been published regarding the preparation of superhydrophobic ZIF-90/sponge composites that were functionalized with eco-friendly short-chain CF_3_ groups for the purpose of selective oil−water separation. Additionally, certain drawbacks of intrinsic powder MOFs, such as potential secondary environmental pollution and difficulty in recovery, limit their practical application in the adsorption and separation of organic compounds [42]. Ongoing efforts are devoted to developing approaches for the conversion of MOFs into macroscopic materials [43,44]. One effective method is to grow MOFs on porous substrates in situ [45,46,47]. It is thus desirable to prepare superhydrophobic and superoleophilic ZIF-90/commercial sponge composites with high adsorption capacities and separation efficiencies by using a green hydrophobic modifier. 

Based on these investigations, the growth of ZIF-90 nanoparticles on porous commercial sponges, as well as the subsequent modification with CF_3_ groups, may be workable in order to obtain superhydrophobic sponge composite materials. The low surface energy CF_3_ groups can significantly reduce surface energy. Meanwhile, the formation of ZIF-90 nanocrystals can increase the sponge surface roughness. The increased surface roughness and the low surface energy may synergistically improve the hydrophobicity [12,13]. In addition, the porous sponge can serve as a robust support for the ZIF nanoparticles, which may solve the recovery issue of MOF powder particles. The polydopamine layer was first introduced on the sponge substrate to provide rich growth sites for ZIF-90 and to maintain the high affinity between ZIF-90 nanoparticles and the sponge substrate [45]. Herein, the F-ZIF-90/melamine formaldehyde (MF) composite sponge was fabricated by growing ZIF-90 on the polydopamine (PDA)-coated MF sponge in situ and subsequently modifying it with 2,2,2-trifluoroethylamine. The F-ZIF-90/PDA-MF composite sponge exhibited superhydrophobicity, superoleophilicity, and good self-cleaning properties. A high absorption capacity for diverse organic liquids was obtained with good reusability. Regardless of the relative oil−water density, continuous oil−water separation was achieved with a separation efficiency of above 99.5% and a flux of up to 7.1 × 10^5^ L m^−2^ h^−1^.

## 2. Results and Discussion

### 2.1. Fabrication and Characterization of F-ZIF-90@PDA-MF Sponge

**Figure 1:**
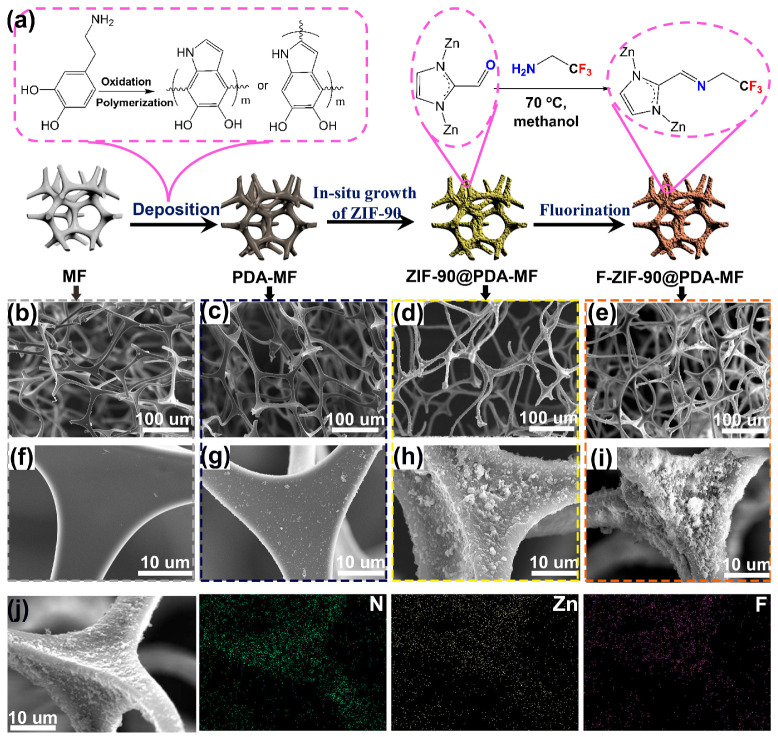
(**a**) Schematic illustration for the fabrication process of F-Zeolitic imidazolate frameworks -90/polydopamine-melamine formaldehyde (F-ZIF-90@PDA-MF). Scanning electron microscope (SEM) images for (**b**,**f**) pristine MF, (**c**,**g**) MF-PDA (**d**,**h**) ZIF-90@PDA-MF and (**e**,**i**) F-ZIF-90@PDA-MF. (**j**) Element mapping images of F-ZIF-90@PDA-MF.

Figure 1a provides a schematic representation of the preparation method for the F-ZIF-90@PDA-MF composite. Firstly, PDA was coated on the MF surface by dopamine self-assembly in a weak alkaline environment. Next, ZIF-90 nanoparticles were grown on the PDA-coated MF sponge in situ, followed by post-synthetic modification via a condensation reaction between the amine groups of TFEA and the aldehyde groups of ZIF-90 [28,39]. Previous reports have confirmed that the coated PDA as the reactive intermediate layer can create a large number of active sites for the subsequent in situ growth of ZIF-90 nanoparticles, and that they can improve the bonding force between ZIF-90 particles and the substrate [45,48,49]. Wang et al. [45] reported that polydopamine provides essential growth sites for ZIF-8 and ensures that ZIF-8 particles adhere firmly to the melamine sponge. In addition, conventional dopamine self-polymerization, with the help of dissolved oxygen, usually takes up to 12 h; in addition, it requires an even longer time to obtain a good PDA coating layer [13,48]. To improve the efficiency of dopamine self-polymerization, NaIO_4_ was selected as a cost-effective oxidation catalyst [50], and the rapid self-polymerization of dopamine was achieved with a coating time of 2 h.

We used a scanning electron microscope (SEM) to examine the surface morphologies of composite sponges. The pristine MF sponge shows porous structures with micrometer-scale pores (Figure 1b) and a clean and smooth surface (Figure 1f) [45,51]. After PDA was coated on the MF surface, there was no noticeable change in the porous network structures (Figure 1c). From a magnified view (Figure 1g), a thin PDA layer was produced on the smooth strut surface. In addition, many particles were attached to the surface, most likely as a result of the self-assembly of superfluous polydopamine aggregations [45,52]. In contrast to the surface of raw MF or PDA-MF, ZIF-90@PDA-MF exhibited a highly rough surface (Figure 1d,h). The increased surface roughness results from the in situ growth of ZIF-90 particles. Porous structures and rough surfaces were retained after ZIF-90 modification (Figure 1e,i). SEM mapping images of F-ZIF-90@PDA-MF (Figure 1j) confirm the homogeneous distributions of Zn and F elements. The two characteristic elements show the uniform decoration of ZIF-90 nanoparticles and the successful modification of ZIF-90 with the low surface energy of TFEA.

**Figure 2:**
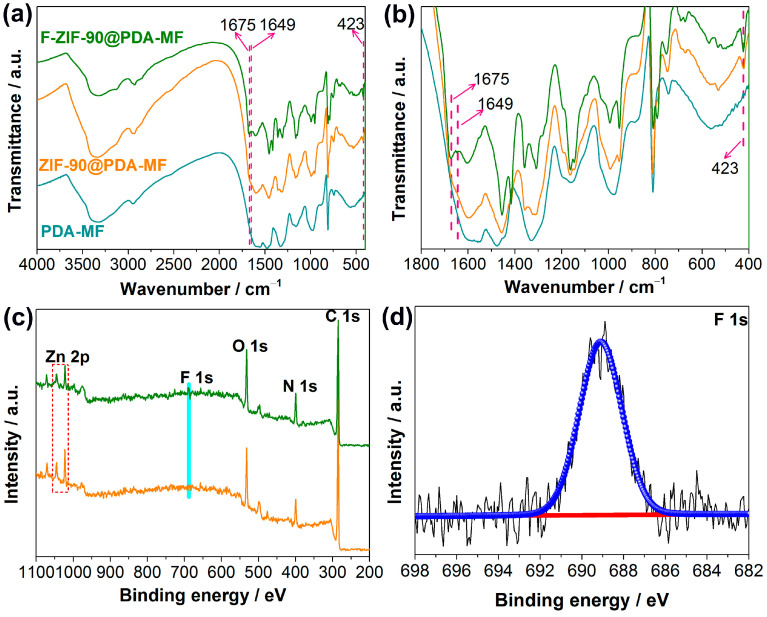
(**a**,**b**) Fourier-transform infrared spectra for PDA-MF, ZIF-90@PDA-MF and F-ZIF-90@PDA-MF composites. (**c**) Wide X-ray photoelectron spectroscopy (XPS) of ZIF-90@PDA-MF and F-ZIF-90@PDA-MF composites and (**d**) high-resolution F 1s XPS spectra of the F-ZIF-90@PDA-MF.

Fourier-transform infrared spectroscopy (FT-IR) analysis was conducted to further show ZIF-90 growth and its post-synthetic modification. The representative peaks of ZIF-90 at 1675 cm^−1^ and 423 cm^−1^ in the FT-IR spectrum of ZIF-90@PDA-MF (Figure 2a) were assigned to C=O [38] and Zn-N [39], respectively. In addition, the vibration peak at 1675 cm^−1^ decreases, and a new peak at 1649 cm^−1^ occurs after the post-synthetic modification of ZIF-90@PDA-MF. This new peak can be ascribed to the formation of C=N [39,40,53,54], which confirms the successful modification via the amine condensation reaction. The FT-IR spectra were explicitly magnified in the region ranging from 400 cm^−1^ to 1800 cm^−1^ for the purpose of clear observation (Figure 2b).

**Figure 3:**
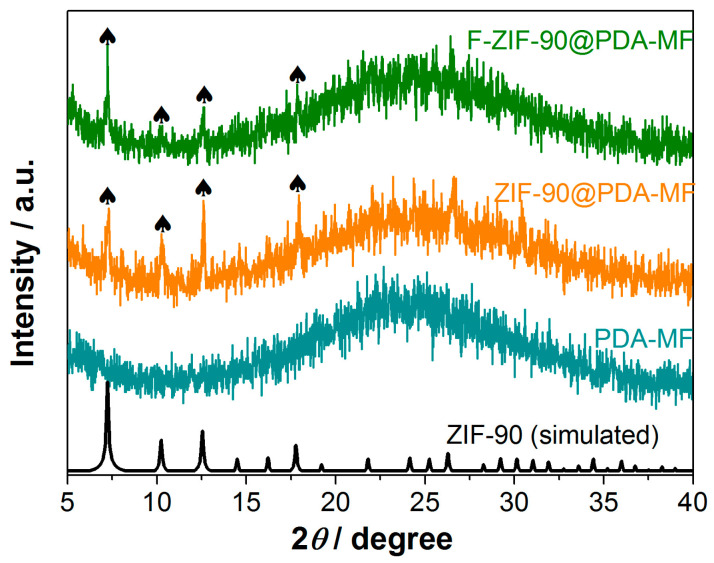
X-ray diffraction patterns for PDA-MF, ZIF-90@PDA-MF, and F-ZIF-90@PDA-MF composite sponges. The black symbol (♠) indicates the main characteristic diffraction peaks of ZIF-90.

The X-ray photoelectron spectroscopy (XPS) survey spectra show that C, N, O, and Zn are the principal elements in the synthesized ZIF-90@PDA-MF and F-ZIF-90@PDA-MF composite sponges (Figure 2c). In addition, a new peak was ascribed to F 1s signal, which occurred in the XPS spectrum of F-ZIF-90@PDA-MF. The F 1s high-resolution spectrum (Figure 2d) shows that a peak at about 689.1 eV corresponds to the highly fluorinated carbon groups [55,56], confirming the presence of CF_3_ groups. This observation further lends support to the successful modification of ZIF-90. As shown in Figure 3, the X-ray diffraction (XRD) pattern of ZIF-90@PDA-MF exhibits the main characteristic diffraction peaks of ZIF-90 at around 7.3°, 10.2°, 12.6°, and 17.9°. The corresponding peaks are in excellent accord with the simulated XRD pattern and literature data [57], thus powerfully demonstrating the effective formation of ZIF-90 nanocrystals. The XRD pattern reveals that the main characteristic peaks were observed after the post-synthetic modification of ZIF-90, thereby suggesting the retention of crystal structures. It is noted that a slight change in the crystallinity and particle morphology of F-ZIF-90 occurred due to the chemical modification (Figure S1 Supplementary Material). Nevertheless, F-ZIF-90 retained the basic crystalline structure of ZIF-90.

### 2.2. Surface Wettability

**Figure 4:**
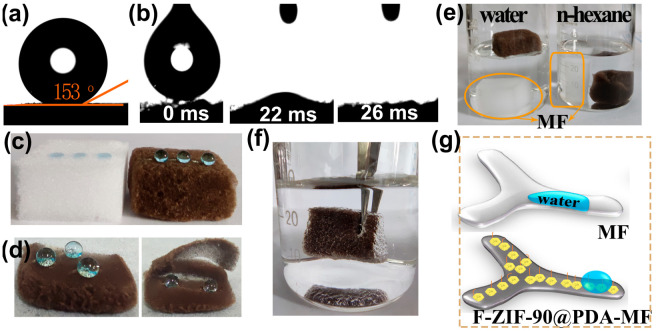
(**a**,**b**) Contact angle images of a water and oil droplet (n-octane) for the F-ZIF-90@PDA-MF composite sponge. (**c**) Water droplets dyed with methylene blue landed on the pristine MF and composite sponge. (**d**) Photographs of dyed water droplets on different zones of the composite sponge: surface (**left**) and cross-section (**right**). (**e**) Photographs for the different wettability of the raw MF sponge and composite sponge in water and n-hexane. (**f**) An image of the composite sponge soaked in water by an external force. (**g**) Schematic description of superhydrophobicity of the F-ZIF-90@PDA-MF sponge.

Contact angle measurements were taken to assess surface wettability properties. MF and PDA-MF sponges both exhibited high hydrophilicity, and water droplets were quickly absorbed (Figure S2a,b); this result agrees with previous reports [58,59]. In contrast, the ZIF-90@PDA-MF composite provides a hydrophobic surface (water contact angle, WCA = 119°, Figure S2c), possibly resulting from the remarkable increment of the surface roughness after the effective formation of ZIF-90 nanocrystals on the PDA-MF substrate (Figure 1h). After modifying ZIF-90@PDA-MF with the CF_3_ groups, a superhydrophobic and superoleophilic composite sponge (i.e., F-ZIF-90@PDA-MF) was obtained with a WCA of 153° and an oil contact angle of about 0° (Figure 4a,b).

Several simple experiments also provide evidence for a noticeable transformation in surface wettability. As displayed in Figure 4c, the raw MF sponge quickly absorbed water droplets that landed on its surface. In contrast, water droplets showed a spherical shape on the composite sponge surface, demonstrating its high hydrophobicity. In addition, spherical water droplets on the superhydrophobic composite sponge could be entirely removed using filter paper (Figure S3). Water droplets exhibit highly spherical shapes on the cross-section and external surface of the composite sponge (Figure 4d), thereby confirming a thorough hydrophobic modification of the bulk sponge. A big difference emerged after the original MF and composite sponge were put in n-hexane and water (Figure 4e). The MF sponge quickly sank to the bottom in both n-hexane and water. However, the F-ZIF-90@PDA-MF floated on the water surface but sank to the bottom in n-hexane. These observations agree with the results of the contact angle tests. In addition, when an external force was used to immerse the composite sponge in water, there were plenty of trapped air bubbles around the sponge surface (Figure 4f). A similar phenomenon has also been widely reported in the literature [50,51]. The changes in the wetting property for superhydrophilic MF and superhydrophobic F-ZIF-90@PDA-MF are schematically illustrated in Figure 4g. It can be explained that the grafted low surface energy of the CF_3_ groups significantly decreases the surface energy and that the ZIF-90 nanocrystals improve the surface roughness [40,45,46]. The hydrophobic performance of solid materials is widely accepted to be dependent on low surface energy and high surface roughness. The synergistic effect of low surface energy and high surface roughness leads to a huge transformation of wetting properties from superhydrophilic to superhydrophobic. Moreover, the stability of the superhydrophobic composite sponge was evaluated under different conditions, including physical squeezing (20 compression-release cycles), low/high temperature (−18 °C and 150 °C for 1 h), and seawater for 1 h. As shown in Figure S4, water drops in spherical shapes occurred on all surfaces after the treatment of superhydrophobic F-ZIF-90@PDA-MF under different conditions. The stability test results suggest that the superhydrophobic F-ZIF-90@PDA-MF can maintain its highly hydrophobic property under different harsh conditions, thus demonstrating its structural stability. 

**Figure 5:**
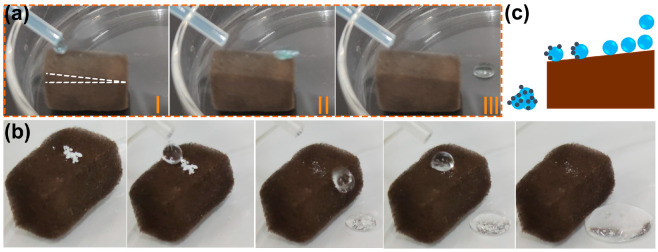
Photographs of (**a**) water-drop rolling test and (**b**) self-cleaning test on the composite sponge. (**c**) Schematic illustration of self-cleaning effect of the superhydrophobic F-ZIF-90@PDA-MF.

It has been demonstrated in previous studies that many superhydrophobic materials have self-cleaning properties [32,41]. Generally, water droplets rolling across the superhydrophobic surface remove impurities that adhere to the material surface. The self-cleaning effect is essential for preventing the contamination of the substrate’s surface in applications. A water drop rolling test was conducted to examine the possibility of self-cleaning on a superhydrophobic F-ZIF-90@PDA-MF composite [60]. As seen in Figure 5a, a series of video shots of a rolling water droplet on the tilted surface (angle < 10°) of the F-ZIF-90@PDA-MF sponge demonstrate that water droplets readily roll away from the tilted F-ZIF-90@PDA-MF sponge. The self-cleaning ability of the superhydrophobic composite sponge was examined by the self-cleaning performance test. White zinc oxide particles were sprinkled on the surface of the composite sponge as grain contaminants [32]. It is clear that due to the rolling of water droplets, the contaminants were taken away from the composite sponge, leaving a relatively clean surface (Figure 5b). Figure 5c schematically depicts the self-cleaning process. It is noted that the small amounts of contaminants that penetrated the composite sponge remained after the end of the self-cleaning test because they could not be contacted by rolling water droplets. Nevertheless, the superhydrophobic composite sponge exhibits promising potential for self-cleaning.

### 2.3. Oil Adsorption and Oil–Water Separation

**Figure 6:**
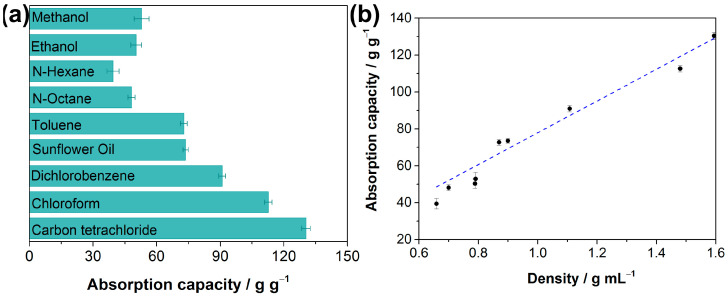
(**a**) Absorption capacities of the F-ZIF-90@PDA-MF sponge toward oil and organic solvents and (**b**) its correlation with different solvent density.

These above results reveal the superhydrophobic and superoleophilic properties of the as-prepared F-ZIF-90@PDA-MF. Oil absorption capacity is generally regarded as one of the more important indexes for absorbent materials to achieve in terms of oil spill recovery [59,61]. Oil adsorption tests were conducted to determine oil absorption capacities of the composite sponge. The adsorption capacity of the as-prepared composite sponge ranged from 39.4 to 130.4 times its weight (Figure 6a), demonstrating that the composite sponge has high absorption capacities for various organic liquids. The oil absorption capacity of the F-ZIF-90@PDA-MF sponge shows relatively good recycling performance and stability. For example, about 88% of its original absorption capacity remains for n-octane even after 20 cycles (Figure S5). In addition, the mass-based absorption capacities were correlated with the liquid density of organic compounds, as plotted in Figure 6b. It was seen that the mass-based absorption capacity of the F-ZIF-90@PDA-MF sponge rose as the liquid density of organic compounds increased. The previous literature reports a similar phenomenon [31,62]. It can be explained that the micron-sized pores of the sponge substrate mainly provide the pore volume for solvent storage [62]. Additionally, as seen in Table 1, the F-ZIF-90@PDA-MF composite sponge achieves comparable or superior water contact angles and absorption capacities of organic compounds with respect to those of various MOF/sponge composite materials.

**Figure 7:**
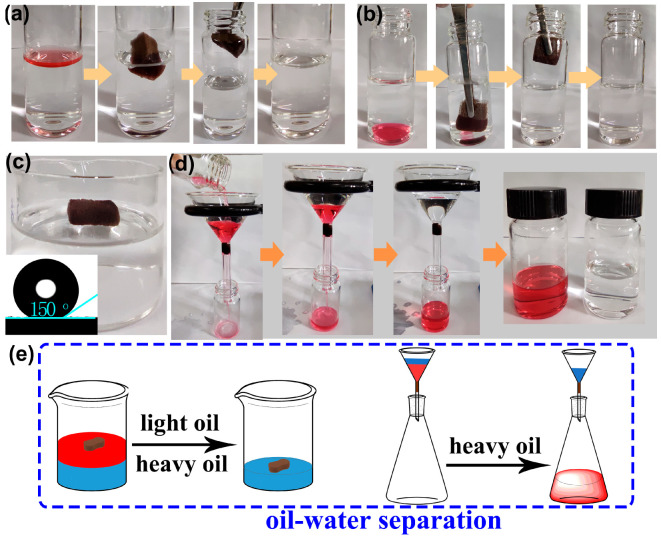
Absorption of (**a**) floating light oil (n-hexane) on the water surface and (**b**) heavy oil (carbon tetrachloride) underwater. (**c**) The F-ZIF-90@PDA-MF composite sponge floated on water after physical squeezing. The inset shows the water contact angle of the composite sponge after 20 absorption-squeezing cyclic tests. (**d**) Heavy oil−water mixture separation by gravity. Carbon tetrachloride and n-hexane were colored with Oil Red O for obvious observation. (**e**) A schematic illustration of batch separation for oil−water mixtures.

Due to the simultaneous superhydrophobic/superoleophilic properties and high oil absorption capacities, it is expected that F-ZIF-90@PDA-MF will show potential use as an absorbent or a filter for efficient oil−water separation [65]. Oil−water separation tests were conducted using the as-prepared composite sponge (Figure 7a,b). We selected carbon tetrachloride and n-hexane as the heavy oil underwater and floating light oil, respectively. Oils were colored with Oil Red O for clear observation. As depicted in Figure 7a,b, the floating n-hexane and carbon tetrachloride underwater were rapidly absorbed when the composite sponge arrived at the oil−water interface. After removing the composite sponge, clean bulk water was observed without red oils. These observations indicate that the superhydrophobic composite sponge has a highly selective adsorption ability for oils from water. For the purposes of 3D oil−water separation materials, using commercial porous sponges as substrates, physical squeezing was a facile and low-cost strategy that could be utilized to collect the adsorbed oil from the saturated adsorbent and to regenerate adsorbents at a laboratory scale [31]. The oil recovery process from the oil-saturated sponge is presented in Figure S6. The F-ZIF-90@PDA-MF still floated on water and the WCA was around 150° even after 20 absorption−squeezing cyclic tests (Figure 7c). Besides, the XRD pattern and SEM image reveal the excellent maintenance of ZIF nanoparticles on the modified sponge (Figure S7). These results indicate the composite sponge shows good cyclic stability under compression.

Gravity-driven separation has previously been reported to be highly efficient and energy-saving [66,67]. To verify the viability of gravity-driven separation, the heavy oil−water mixture system was tested in a simple separation device using the sponge composite as a filter. As shown in Figure 7d, the superhydrophobic composite sponge was fixed as a filter in the neck of a funnel [68]. We selected Oil Red O-stained carbon tetrachloride as a heavy oil underwater. When the heavy oil−water mixture was poured into the funnel, the heavy oil quickly permeated into the composite sponge and naturally flowed into the collection container. In contrast, water was repelled and retained at the top of the funnel. In addition, the water content of the collected carbon tetrachloride was determined, indicating that the separation efficiency reached 99.9%. The simultaneous superhydrophobicity and superoleophilicity of F-ZIF-90@PDA-MF guarantee a highly selective separation. Figure 7e succinctly summarizes the bath separation for light oil/heavy oil−water mixtures, using the F-ZIF-90@PDA-MF composite sponges as an absorbent or a filter.

### 2.4. Continuous Oil–Water Separation

**Figure 8:**
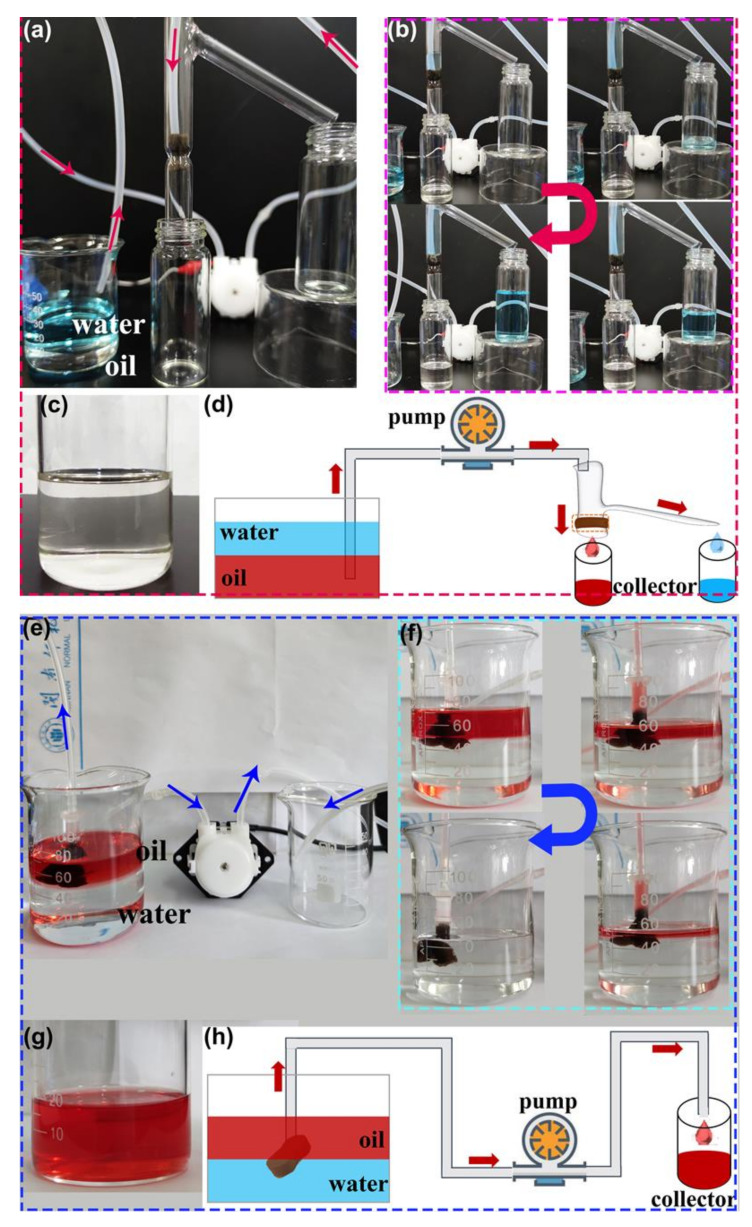
Photographs of (**a**,**e**) the continuous oil/water separation system, (**b**,**f**) the progress of the continuous removal of oils (carbon tetrachloride and n-hexane, respectively) from the water. (**c**,**g**) Photographs of the collected oils. (**d**,**h**) Schematic summary of continuous separation for oil−water mixtures.

Continuous oil−water separation is highly needed, especially for large-scale separation. Continuous separation experiments were carried out in designed separation systems in order to expand the practical application in the separation of vast volumes of the oil−water mixture (Figure 8a,e). The superhydrophobic composite sponge serves as a filter and an absorbent in Figure 8a,e, respectively. 

As discussed above, gravity makes it simple to achieve the bath separation of heavy oil−water mixtures (Figure 7d). If water can be immediately removed from the top of the separation device, then gravity-driven continuous separation is accomplished. As presented in Figure 8a, a simple separation device with two outlets was designed and applied in the continuous separation of the heavy oil−water mixture. The as-prepared F-ZIF-90@PDA-MF sponge composite, serving as a filter, was fixed in the separation device. When the continuous flow of heavy oil−water mixture entered the separation device and came into contact with the superhydrophobic and superoleophilic composite sponge, the colorless and transparent heavy oil quickly penetrated the composite sponge and smoothly flowed to the oil collecting container at the bottom (Figure 8b). At the same time, the dyed water was repelled and kept in the upper space and flowed into a water-collecting container. This result demonstrates the viability of gravity-driven continuous separation in the homemade separation device.

The majority of the reported hydrophobic sponge-based adsorbents have a high oil absorption capacity of tens of their weight [7]. In order to use adsorbent materials for the subsequent cycle, the absorbed oil needs to be promptly removed from the absorbent when the absorbent is saturated [7,65]. Based on this analysis, as shown in Figure 8e, another separation device for continuous oil−water separation was tested. The composite sponge, as an absorbent, was fixed in the tube and connected with an external self-priming pump. When the superhydrophobic and superoleophilic composite sponge was brought into contact with the oil/water interface, the oil molecules easily permeated into the free space in the composite sponge and were then locked in the sponge. In contrast, the water molecules were strongly repelled. Once the absorbed oil in the sponge was transported into the collector via the tube as soon as the pump was turned on, the freed-up pore space could further absorb the additional floating oil. As a result, the continuous collection of oil was achieved. Figure 8f shows the continuous separation process of the light oil−water mixture. The removal and collection of oils from the water was successfully accomplished by F-ZIF-90@PDA-MF with the aid of a pump. Furthermore, as shown in Figure 8c,g, no water droplets in the collected oil were visible to the naked eye, thereby indicating a highly selective separation. A schematic illustration of the two aforementioned separation models is presented in Figure 8d,h. In brief, these experimental results demonstrate efficient continuous separation of oil and water. 

**Figure 9:**
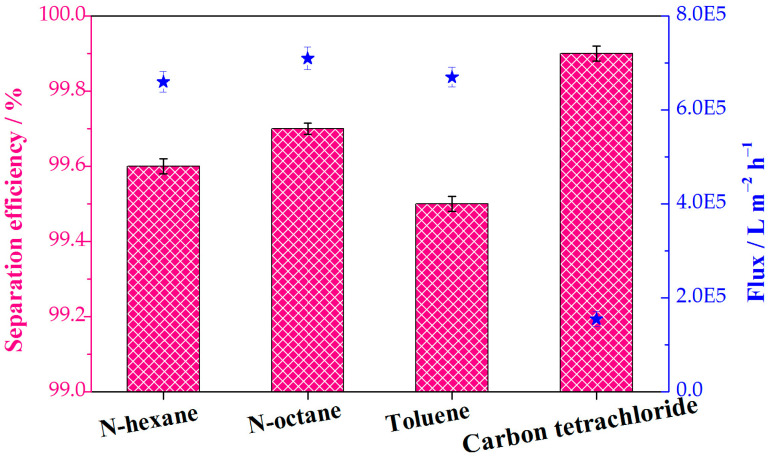
The separation efficiency and flux of various immiscible oil−water mixtures in the continuous separation process. The pink columns and blue stars represent the separation efficiency and flux, respectively.

The water contents of the collected oils were quantified to further determine the separation efficiencies of the continuous separations. Various immiscible oil−water mixtures (n-hexane water, n-octane water, toluene water, and carbon tetrachloride water) were selected in order to investigate the separation efficiency and flux. Separation efficiency values for all tested oil−water mixtures exceeded 99% (Figure 9). This result proves the almost entire separation of oils from water and the feasibility of continuous oil−water separation. The superhydrophobic composite sponge showed a high flux for the various oil−water system, which is 1.55 × 10^5^ L m^−2^ h^−1^ and more than 6.01 × 10^5^ L m^−2^ h^−1^ for the gravity-driven continuous separation and pump-assisted absorption separation, respectively. In addition, the flux for the continuous separation is comparable and/or superior to those of reported MOF-based sponges and membranes (Table S1). The porous structures of the sponge substrate and the external separation drive force provided by a pump are credited with the high flux.

## 3. Materials and Methods

### 3.1. Materials

Tris(hydroxymethyl)-aminomethane (Tris, 99.8%), imidazole-2-formaldehyde (98%), and the 2,2,2-trifluoroethylamine (TFEA, 99%) were received from Aladdin Company. Zinc nitrate hexahydrate (99%) and chloroform were purchased from Xilong Scientific Co., Ltd., Guangdong, China. The various analytical grade solvents (methanol, n-hexane, n-octane, carbon tetrachloride, toluene, and chlorobenzene), Oil Red O (biological stain), methylene blue (biological stain), sodium formate (99.5%), sodium metaperiodate (99.5%), and dopamine hydrochloride (98%) were purchased from Innochem Technology Co., Ltd., Beijing, China. All chemicals were used as received. Both sunflower oil and melamine formaldehyde (MF) sponges were supplied as commercial products. 

### 3.2. Preparation of PDA-Coated MF Sponge

A polydopamine-coated MF (PDA-MF) sponge was prepared based on a previously described process with slight modifications [50,69]. Dopamine hydrochloride (2 mg mL^−1^) was mixed in a solution of 10 mM Tris-HCl (pH = 8.5) containing 2 mM of NaIO_4_. Subsequently, the pristine MF sponge was immersed in the above mixture and stirred vigorously at an ambient temperature for 2 h. The as-prepared PDA-MF material was dried in a vacuum at 80 °C after being washed with water and ethanol.

### 3.3. Preparation of F-ZIF-90@PDA-MF Sponge

A mixture of zinc nitrate hexahydrate (1 mmol), imidazole-2-formaldehyde (4 mmol), and sodium formate (1 mmol) was dissolved in 30 mL of methanol by stirring and ultrasonic treatment in order to obtain a homogeneous solution. The as-prepared PDA-MF sponge was soaked in the solution. The mixture was transferred into a 50 mL Teflon-lined steel autoclave and heated at 85 °C for 24 h. At the end of the solvothermal treatment, the composite sponge was washed with methanol three times and dried overnight in a vacuum at 80 °C. The obtained composite sponge was referred to as ZIF-90@PDA-MF. The F-ZIF-90@PDA-MF sponge was obtained by fluorination of the ZIF-90@PDA-MF sponge via an amine condensation reaction [28]. Typically, ZIF-90@PDA-MF was placed in a methanol solution of TFEA (0.15 M) and refluxed for 24 h at 70 °C. After the reaction finished, the sponge was taken out and thoroughly washed with fresh methanol three times to remove the residual free TFEA molecules. Finally, the obtained F-ZIF-90@PDA-MF was dried in a vacuum at 80 °C.

### 3.4. Characterization of Samples

The morphology and elementary chemical compositions were obtained on a scanning electron microscope (JEOL JSM-6010LA), which was combined with energy-dispersive X-ray microanalysis. The X-ray diffraction patterns were collected on an X-ray diffractometer (Rigaku Ultima IV) with Cu-Kα emission. The data for X-ray photoelectron spectroscopy were obtained using an X-ray photoelectron spectrometer with an Al Kα line source (Thermo Scientific Escalab Xi +,Waltham, MA, United States). The binding energies were calibrated at the C 1s signal of 284.8 eV. Fourier-transform infrared spectra were collected on a spectrometer (Thermo Nicolet iS-10) in the range of 4000–400 cm^−1^, with a resolution of 4 cm^−1^ and with 32 scans. The contact angle was measured with a 4 μL droplet on a contact angle instrument (Dataphysics OCA20) under ambient conditions. The water content was measured by using an MKC-610 Karl Fischer moisture titrator.

### 3.5. Oil Adsorption

Composite sponges were immersed in organic oily liquids at room temperature for 30 s. Liquid-saturated sponges were removed and drained for a few seconds in air, followed by quick weighing. According to the following Equation (1), the oil absorption capacity (*q,* g g^−1^) was determined by the mass of the absorbent both before and after oil adsorption. Each value was averaged from three parallel experiments.

*q* = (*m*_1_ − *m*_0_)/*m*_0_ (1)

where *m*_0_ and *m*_1_ are the masses of the composite sponge, both before and after absorbing oil or organic solvents.

### 3.6. Oil–Water Separation Experiments

The continuous separation of oil–water mixtures was tested on home-made separation devices. The F-ZIF-90@PDA-MF, serving as an absorbent and/or the filter, was fixed in the separation device. Various oil–water mixtures were obtained by simply mixing water and the Oil Red O-stained oils with a volume ratio of 2.5:1. The separation efficiency (*E*) was determined based on the following Equation (2) [59]:

*E* = (1 − *C*_f_/*C*_0_) × 100% (2)

where *C*_0_ and *C*_f_ are the water content in the initial water–oil mixtures and the collected oil, respectively.

## 4. Conclusions

In this work, a green modification method using eco-friendly short fluorine-carbon chains was developed to prepare the superhydrophobic/superoleophilic ZIF-90/commercial sponge composites with a high adsorption capacity and separation efficiency for continuous oil–water separation. The superhydrophobic F-ZIF-90@PDA-MF composite was successfully fabricated by growing ZIF-90 particles in situ on the PDA-coated MF substrate, followed by the post-synthetic modification of ZIF-90 with trifluoroethylamine. The as-synthesized composite sponge shows simultaneous superhydrophobicity and superoleophilicity with a WCA of 153° and good self-cleaning performance. The rough surface produced by the in situ formation of ZIF-90 particles and the low surface energy that was brought on by the grafted trifluoroethylamine containing CF_3_ groups are both responsible for the superhydrophobicity. As a result of superhydrophobicity/superoleophilicity, the composite sponge exhibits good absorption performance with high sorption selectivity for organic compounds from water. The high adsorption capacity ranged from 40 to 130.4 g g^−1^ for different organic compounds, which is correlated with the density of organic compounds. Importantly, the adsorbed organic compounds were quickly recovered by simple physical squeezing. Moreover, after 20 absorption−squeezing cycles, the composite sponge retains its superhydrophobicity with a WCA of 150°. The bath separation and continuous separation of the various oil/water mixture systems were realized. The separation efficiency and flux can reach above 99.5% and up to 7.1 × 10^5^ L m^−2^ h^−1^, respectively. These results show that the superhydrophobic and superoleophilic F-ZIF@PDA-MF composite sponge has the potential to solve large-scale organic pollution in a water environment. This research offers a simple method to design superhydrophobic MOF-based sponge materials using alternative short-chain fluorinated substances for the efficient purification of wastewater.

## Figures and Tables

**Table 1:** Comparison of oil absorption capacities and water contact angles of various metal−organic frameworks (MOF)/sponge composite materials.

MOF/Sponge Sorbents	Water Contact Angle/°	Oil Absorption Capacity /g g^−1^	Ref.
ZIF-8@rGO@PU	171	14–29	[63]
MF-ZIF-8	140	10–38	[45]
ZIF-POSS@PDA@PU	--	19–40	[57]
ZIF-90-CF_3_/MF	132	40.1–108.7	[40]
SA@ZIF-8@PU	140.8	30.3–115.4	[64]
ZIF-8/MF	130	76–164	[59]
Zr-BDC-OH@CF3@MF	145	27–47	[41]
UiO-66(COOH)2/PU	161	29–56	[25]
MIL-DDT@MF	151.8	54.1–120.2	[13]
MS-CMC-HPU-13	127	66–130	[24]
F-ZIF@PDA-MF	153	39.4–130.4	This work

## Data Availability

Data available on request. The data presented in this study are available on request from the corresponding author.

## Supplementary Materials

The following supporting information can be downloaded at: https://www.mdpi.com/article/10.3390/molecules28062843/s1. Figure S1: XRD and SEM data for pure ZIF-90 and F-ZIF-90 powder particles; Figure S2: (a–c) Water contact angle images of the pristine MF, PDA-MF, ZIF-90@PDA-MF, respectively; Figure S3: The removal of water droplets on the surface of F-ZIF-90@PDA-MF by using the filter paper; Figure S4: Water droplets on the surface of the F-ZIF-90@PDA-MF sponge after exposure to different conditions; Figure S5: Recyclability experiments for the absorption capacity of n-octane by the F-ZIF-90@PDA-MF sponge; Figure S6: A physical squeezing method for oil recovery from the oil-saturated F-ZIF-90@PDA-MF absorbent and absorbent regeneration; Figure S7: (a) XRD and (b) SEM for the composite sponge after the cyclic experiment of oil–water separation; Table S1: Comparisons of various MOF-based materials for oil−water separation [70,71,72].

## Sample Availability

Samples of the compounds are available from the authors.
