# Supplementary material for: Eco-Friendly Fluorine Functionalized Superhydrophobic/Superoleophilic Zeolitic Imidazolate Frameworks–Based Composite for Continuous Oil–Water Separation
Source: Molecules. 2023 Mar 21;28(6):2843. doi: 10.3390/molecules28062843 (PMC10054728; doi:10.3390/molecules28062843)
Supplement: Supplementary file 1 [file molecules-28-02843-s001.zip › molecules-2261342-supplementary.pdf]

Supporting Information

Eco-Friendly Fluorine Functionalized Superhydrophobic/Superoleophilic Zeolitic Imidazolate Frameworks−Based Composite for Continuous Oil−Water Separation

Wenlong Xiang [1,2,*], Siyu Gong [1] and Jiabin Zhu [1]

[1]*College of Chemistry, Chemical Engineering and Environment, Minnan Normal University, Zhangzhou 363000, China*

[2] *Fujian Province University Key Laboratory of Pollution Monitoring and Control, Minnan Normal University, Zhangzhou 363000, China*

* Corresponding author

E-mail address: wenlong_xiang@126.com; wenlongx2290@mnnu.edu.cn

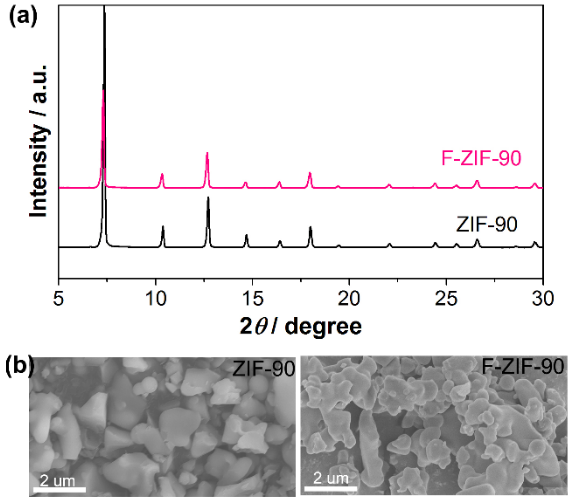

Figure S1. XRD and SEM data for pure ZIF-90 and F-ZIF-90 powder particles.

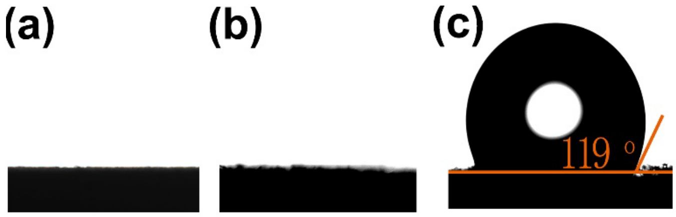

Figure S2. (**a**-**c**) Water contact angle images of the pristine MF, PDA-MF, ZIF-90@PDA-MF, respectively.

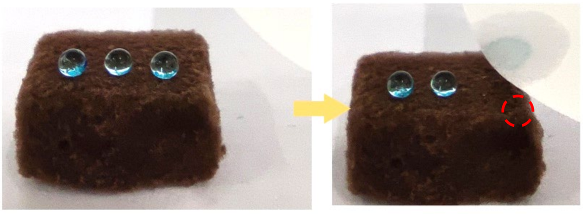

Figure S3. The removal of water droplets on the surface of F-ZIF-90@PDA-MF by using the filter paper.

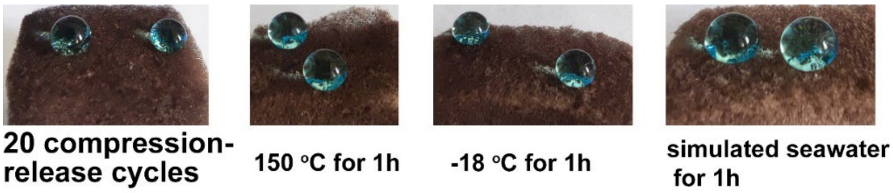

Figure S4. Water droplets on the surface of the F-ZIF-90@PDA-MF sponge after exposure to different conditions.

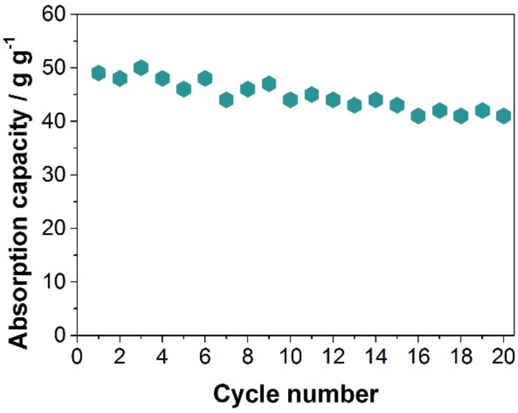

Figure S5. Recyclability experiments for the absorption capacity of n-octane by the F-ZIF-90@PDA-MF sponge.

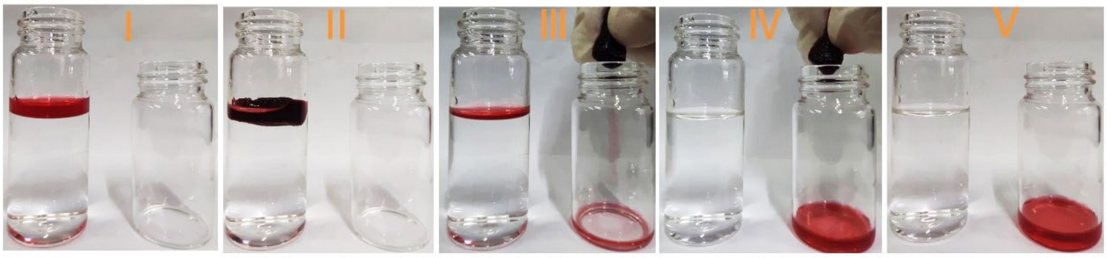

Figure S6. A physical squeezing method for oil recovery from the oil-saturated F-ZIF-90@PDA-MF absorbent and absorbent regeneration

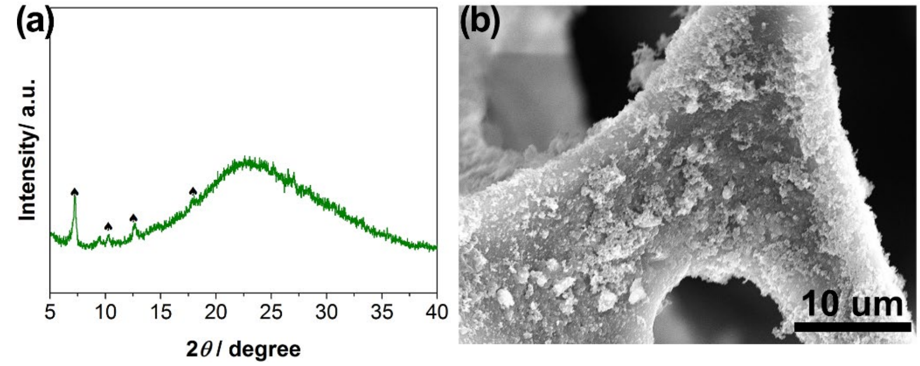

Figure S7. (a) XRD and (b) SEM for the composite sponge after the cyclic experiment of oil−water separation

Table S1. Comparisons of various MOF-based materials for oil–water separation.

Materials	Separation driving force	Separation efficiency / %	Flux [1] / L m^{-2} h^{-1}	Ref.
ZIF-8@GSH/PI membrane	gravity	>99	5625	[70]
UiO-66-F4@rGO/FP	gravity	>99	990	[71]
UiO-66-F4@rGO/MS	pump-assisted	n.d.	n.d.	[71]
Zr-BDC-OH@CF_3@MS	gravity	98.5	2028	[41]
UiO-66-$(COOH)_2$-PU	gravity/ pump-assisted	96	n.d.	[25]
Co-ZIF-L@MS	gravity	99.7	2.82×10^5	[72]
PDMS-Fe_3O_4@MF	gravity	99.9	1.35×10^5	[50]
ZIF-8/MF	gravity	99.9	1.63×10^5	[59]
F-ZIF-90@PDA-MF	gravity	99.9	1.55×10^5	**This work**
	pump-assisted	99.5	7.1×10^5	

[1] maximal flux reported in literature.

n.d.: not determined
